# Assessment of feline peripheral blood lymphocyte subpopulations and CD18 expression pattern by flow cytometry

**DOI:** 10.29374/2527-2179.bjvm006125

**Published:** 2025-12-17

**Authors:** Patricia Lindiman, Rosina Sánchez Solé, Paula Pessina, María Florencia Mosquillo

**Affiliations:** 1 Laboratorio de Análisis Clínicos, Endocrinología y Metabolismo Animal, Departamento de Clínicas y Hospital Veterinario, Facultad de Veterinaria, Universidad de la República, Montevideo, 13000, Uruguay.; 2 Instituto de Investigación Una Salud, Universidad de la República, Montevideo, 11200, Uruguay.

**Keywords:** flow cytometry, immunophenotyping, pan-leukocyte marker, citometria de fluxo, imunofenotipagem, marcador pan-leucocitário

## Abstract

Flow cytometry-based immunophenotyping plays a critical role in the diagnosis, classification, staging, and monitoring of hematopoietic malignancies. However, its application in feline medicine remains limited primarily because of the scarcity of species-specific antibodies. Existing studies in cats predominantly focus on analyzing lymphocyte subpopulations in lymph nodes, with fewer investigations conducted on bone marrow and peripheral blood. This study aimed to assess the utility of peripheral blood samples from healthy cats for quantifying lymphocyte subpopulations by flow cytometry to provide basic data useful for future studies on animals with diseases. Using specific antibody panels, we successfully quantified lymphocyte subpopulations in 15 healthy cats. Total leukocytes (CD18+) accounted for a median of 89.7% of peripheral blood cells. Among lymphocytes, CD5+ T cells were the predominant subset, followed by CD21+ B cells. Among T cells, CD4+ helper cells outnumbered CD8+ cytotoxic cells. Notably, CD18 expression exhibited a biphasic pattern: B lymphocytes showed lower fluorescence intensity compared with T lymphocytes. In some cases, three distinct fluorescence peaks suggested further heterogeneity within the T-cell population. To the best of our knowledge, this is the first study to clearly identify a biphasic expression pattern of CD18 in the peripheral blood lymphocytes of cats. These findings reveal the complexity of immune cell marker expression in the peripheral blood of felines and support the broader application of flow cytometry in feline immunological and diagnostic research. The establishment of these baseline immunophenotypic profiles in healthy cats is crucial for improving the diagnosis and monitoring of feline hematopoietic diseases, ultimately contributing to better clinical management and therapeutic strategies.

## Resumo

A imunofenotipagem baseada em citometria de fluxo desempenha um papel fundamental no diagnóstico, classificação, estadiamento e monitoramento de malignidades hematopoéticas. No entanto, sua aplicação na medicina felina permanece limitada, principalmente devido à escassez de anticorpos específicos para a espécie. Estudos existentes em gatos focam predominantemente na análise de subpopulações de linfócitos em linfonodos, com menos investigações realizadas na medula óssea e no sangue periférico. Este estudo teve como objetivo avaliar a utilidade de amostras de sangue periférico de gatos saudáveis para quantificar subpopulações de linfócitos por citometria de fluxo, a fim de fornecer dados básicos úteis para futuros estudos em animais com doenças. Utilizando painéis específicos de anticorpos, quantificamos com sucesso subpopulações de linfócitos em 15 gatos saudáveis. Os leucócitos (CD18+) representaram uma mediana de 89,7% das células do sangue periférico. Entre os linfócitos, as células T CD5+ foram o subconjunto predominante, seguidas pelas células B CD21+. Dentro das células T, as células auxiliares CD4+ foram mais numerosas que as citotóxicas CD8+. Notavelmente, a expressão de CD18 apresentou um padrão bifásico: os linfócitos B mostraram intensidade de fluorescência mais baixa em comparação com os linfócitos T. Em alguns casos, três picos distintos de fluorescência sugeriram maior heterogeneidade dentro da população de células T. Até onde sabemos, este é o primeiro estudo a identificar claramente um padrão de expressão bifásica de CD18 em linfócitos do sangue periférico de gatos. Esses achados revelam a complexidade da expressão de marcadores celulares imunes no sangue periférico felino e apoiam a aplicação mais ampla da citometria de fluxo na pesquisa imunológica e diagnóstica felina. O estabelecimento desses perfis imunofenotípicos básicos em gatos saudáveis é crucial para melhorar o diagnóstico e monitoramento das doenças hematopoéticas felinas, contribuindo, em última instância, para uma melhor gestão clínica e estratégias terapêuticas.

## Introduction

Flow cytometry is an advanced analytical technology that enables multiparametric quantitative analysis of the physical and biochemical properties of individual cells or particles in suspension by measuring light scattering and fluorescence emissions as they pass through one or more laser beams in a fluid stream (Manohar et al., 2021). This technique facilitates rapid immunophenotyping, cell cycle analysis, and functional assays at the single-cell level and has a wide range of applications (McKinnon, 2018).

Currently, flow cytometry is a crucial tool in both human and veterinary medicine for diagnosing and monitoring hematopoietic diseases. The analysis of peripheral blood lymphocyte subsets contributes to the diagnosis and monitoring of hematopoietic malignancies by identifying abnormal or clonal populations, distinguishing neoplastic from reactive lymphocytosis, and enabling minimally invasive disease monitoring (Béné et al., 2016). Lymphomas and leukemias usually involve the clonal expansion of specific lymphocyte subsets, and flow cytometry facilitates their identification through the detection of phenotypic abnormalities or skewed subset distributions, aiding in diagnosis and classification (Rout et al., 2020). Additionally, lymphocyte subset profiles also reflect patterns of immune response, where alterations in the CD4/CD8 ratio and expression of activation markers can indicate immune activation, suppression, or dysregulation associated with infections, chronic inflammation, or immunodeficiency (Bęczkowski et al., 2015). These approaches improve diagnostic accuracy and aid in the clinical management of veterinary patients.

Recently, the application of flow cytometry has proven particularly valuable in the diagnosis of lymphoproliferative and myeloproliferative diseases in dogs, prompting the need for its further development across different animal species (Evans, 2023). Our group has successfully applied flow cytometry for the immunophenotypic characterization of canine lymphomas and leukemias, contributing to improved diagnostic accuracy, disease classification, and treatment guidance (Sánchez-Solé et al., 2021, 2022). One of the primary challenges in extending flow cytometry to cats lies in the limited availability of species-specific antibodies. Consequently, assays often rely on antibodies developed for humans or dogs, with little information on their cross-reactivity across species (Araghi et al., 2014). In felines, the main studies have investigated lymphocyte subpopulations in normal, slightly reactive or neoplastic lymph nodes and, to a lesser extent, in the bone marrow (Araghi et al., 2014; Bęczkowski et al., 2015; Byrne et al., 2000; Campbell et al., 2004; Comazzi et al., 2006; Hofmann-Lehmann et al., 1997; Martini et al., 2018, 2020; Rout et al., 2020; Rütgen et al., 2022; Shirani et al., 2011; Vapniarsky et al., 2020; Willett et al., 1993). However, studies involving mononuclear cells from peripheral blood remain scarce.

Although still in its early stages for feline species, initial studies have examined preanalytical parameters by flow cytometry using lymph node samples from cats with lymphoma (Martini et al., 2018). Additionally, the expression patterns of several pan-leukocyte markers, such as CD44 and CD18, have been explored in the peripheral blood of healthy cats (Martini et al., 2020). Specifically, anti-CD18 was used to assess the proportion of leukocytes present, while anti-CD5 was used to identify T lymphocytes. Furthermore, anti-CD4 and anti-CD8 antibodies allowed discrimination between helper and cytotoxic T-cell subsets, respectively, and anti-CD21 antibodies aided in the characterization of B-cell lymphomas. Together, this antibody combination enabled a comprehensive profiling of the cellular components in blood samples. CD18, an adhesion molecule (integrin β2), is expressed on the surface of all white blood cell subclasses, with expression levels varying according to the state of cell activation; its expression is typically increased in activated cells (Comazzi et al., 2006). Moreover, higher CD18 expression has been reported in monocytes and polymorphonuclear cells than in lymphocytes (Comazzi et al., 2006; Martini et al., 2020).

In this context, the present study aimed to quantify lymphocyte subpopulations in healthy cats (B lymphocytes, T helper lymphocytes and T cytotoxic lymphocytes) and to investigate the differential expression patterns of the pan-leukocyte marker CD18 within the lymphocyte population under the hypothesis that flow cytometric analysis of feline peripheral blood would reveal distinct patterns of CD18 expression, providing useful baseline data for future comparative studies in diseased animals and potentially contributing to diagnostic applications.

## Materials and methods

### Animals and samples

The lymphocyte marker expression of 15 cats was analyzed. All the animals were obtained from private veterinary clinics and the Faculty of Veterinary Medicine of the University of the Republic of Uruguay. The cats included in this study were healthy, of undefined breed, of both sexes, and aged between 8 months and 12 years (median: 4 years) (Table 1). All the animals underwent clinical and physical examinations and had no previous history of disease prior to sample collection. Laboratory tests were performed, including complete blood count, blood chemistry, and serology for FIV and FeLV, which were normal in all cases. For immunophenotyping by flow cytometry, a 0.5 mL blood sample was drawn from the cephalic vein of all the study animals using 21G needles and placed in tubes containing ethylenediaminetetraacetic acid (EDTA). All the samples were stored at 4 °C until processing.

**Table 1:** The demographic data of the 15 healthy cats were analyzed, including identification number, sex, age, and breed.

**ID**	**Sex**	**Age (years)**	**Breed**
1	Female	4	Mixed
2	Male	12	Mixed
3	Male	3	Mixed
4	Male	8	Mixed
5	Female	4	Mixed
6	Male	5	Mixed
7	Female	11	Mixed
8	Female	0.7	Mixed
9	Female	1	Mixed
10	Male	0.8	Mixed
11	Male	7	Mixed
12	Female	9	Mixed
13	Male	0.8	Mixed
14	Female	7	Mixed
15	Male	3	Mixed

### Flow cytometry

Flow cytometry analysis of peripheral blood samples from felines was performed on an Accuri C6 flow cytometer (BD Biosciences, CA). The staining protocol was performed on peripheral blood samples with EDTA stored at 4 °C within 24 hours after the sample was obtained to avoid cell death (Martini et al., 2018). Before the blood was aliquoted, it was homogenized in a shaker for 2 minutes. A total of 25 µL of peripheral blood was used in each undiluted tube, and to evaluate cell viability, the cells were stained with 20 µg/mL propidium iodide (PI; Thermo Fisher Scientific). Immediately, red blood cell lysis was performed using the BD PharmLyse kit, following the manufacturer's recommendations. Briefly, a 10:1 ratio of lysis buffer:sample was used and incubated for 15 minutes in the dark. Once the lysis was completed, the washing procedure was continued at the same 10:1 ratio, using PBS-FBS buffer (1X phosphate-buffered saline (PBS) supplemented with 1% fetal bovine serum (FBS) inactivated at 56 °C for 30 minutes and 0.1% sodium azide). After centrifugation (200 g, 5 minutes), the samples were placed in PBS-FBS buffer in a final volume of 100 µL. Only samples with a viability greater than 85% were included in the study.

From the available antibodies, 3 panels were designed to analyze white blood cells (Table 2). A panel of directly conjugated antibodies—including fluorescein isothiocyanate (FITC), phycoerythrin (PE), and Alexa Fluor 647—was used to analyze antigen expression and determine the immunophenotype of the samples. The optimized staining process consisted of incubating the antibodies (1:10 dilution according to the manufacturer’s instructions) for 30 minutes in the dark at room temperature while keeping the tubes on a shaker. Next, red blood cell lysis was performed using the BD PharmLyse kit, as described below. The samples were vortexed after the addition of antibodies, and after each incubation, washes were performed, as well as before acquisition on the cytometer.

**Table 2:** List of monoclonal antibodies used for flow cytometric phenotyping of peripheral blood lymphocyte subpopulations, including clones, fluorescent labels, sources and target species.

**Monoclonal antibody**	**Fluorochrome**	**Specificity**	**Target species**	**Clone**	**Source**	**References**
**Panel 1**						
CD4	Fluorescein isothiocyanate (FITC)	T helper lymphocyte	Feline	vpg34	BIO-RAD	Campbell et al. (2004)
CD8α/β	Phycoerythrin (PE)	T cytotoxic lymphocyte	Feline	vpg9	BIO-RAD	Campbell et al. (2004)
CD18	Alexa Fluor 647	Pan-leukocyte	Canine	CA1.4E9	BIO-RAD	Comazzi et al. (2006); Rütgen et al. (2022)
**Panel 2**						
CD5	Fluorescein isothiocyanate (FITC)	T lymphocyte	Feline	FE1.1B11	BIO-RAD	Knotek et al. (2000)
CD21	Alexa Fluor 647	B lymphocyte	Canine	CA2.1D6	BIO-RAD	Rout et al. (2020); Vapniarsky et al. (2020)
**Panel 3**						
CD18	Alexa Fluor 647	Pan-leukocyte	Canine	CA1.4E9	BIO-RAD	Comazzi et al. (2006); Rütgen et al. (2022)
CD5	Fluorescein isothiocyanate (FITC)	T lymphocyte	Feline	FE1.1B11	BIO-RAD	Knotek et al. (2000)
CD21	Phycoerythrin (PE)	B lymphocyte	Canine	CA2.1D6	BIO-RAD	Martini et al. (2020)

For data acquisition from peripheral blood samples, a dot plot of forward scatter (FSC) *vs.* side scatter (SSC) was generated. A debris threshold was set to eliminate events with low forward and side scatter properties (threshold of 800,000 in forward scatter height, FSC-H). In addition, a plot was constructed to eliminate doublets using the forward scatter area (FSC-H *vs.* FSC-A) so that only singletons were considered. All samples were acquired in slow mode, and a minimum of 5000 lymphocytes were acquired. Parameter analysis was performed using a bandpass filter of 533/30 nm for FITC-conjugated antibodies (fluorescence channel 1 (FL1)), 585/40 nm for PE-conjugated antibodies (FL2), and 675/25 nm for the Alexa 647 fluorophore (FL4). Single-staining controls were used to calculate color compensation. The FL1 detector was corrected by subtracting a percentage of 4.3% from FL2, and the compensation value for the FL2 channel was 9.1%, which was deducted from FL1. To identify the different leukocyte subpopulations, a backgating strategy was used, which is a method that allows the analysis of gates in dot plots of interest, such as fluorescence *vs.* SSC, and once a region with particular characteristics was identified, it was retrofitted in the FSC *vs.* SSC plot to confirm the population. Each sample was analyzed with an unlabeled tube (negative control), and the same procedure was performed. In addition, fluorescence minus one (FMO) controls were used to define the cutoff point for the fluorescence intensity of the positive and negative populations, which was set for all the determinations. The data were analyzed with C6 Plus Analysis software (BD Biosciences, CA). For each variable, descriptive data were summarized using the median and the minimum–maximum range.

## Results

### Expression values of surface markers on feline lymphocytes

Using Panel 1 (CD18-Alexa Fluor 647, CD4-FITC, and CD8-PE) and Panel 2 (CD21-Alexa Fluor 647 and CD5-FITC), specific labeling of lymphocyte surface molecules was successfully achieved through antibody staining. This enabled the quantification of total lymphocytes and their respective subsets in a cohort of 15 cats. The median percentage of viable leukocytes (IP−, CD18+) in peripheral blood was 89.7%. Among the lymphocyte subpopulations, CD5+ T lymphocytes represented the majority (47.1%), followed by CD21+ B lymphocytes (29.5%). Within the T-cell compartment, CD4+ helper T cells were more prevalent (25.3%) than CD8+ cytotoxic T cells were (15.3%), resulting in a variable CD4/CD8 ratio across individuals, which ranged from 0.8 to 3.3, with a median of 1.3. The distribution and percentage of each lymphocyte subset are detailed in Table 3.

**Table 3:** Lymphocyte subpopulations in the peripheral blood of 15 healthy cats.

**ID**	**Viable cells (PI-)**	**CD18+**	**CD21+**	**CD5+**	**CD4+**	**CD8+**	**CD4+/CD8+**
1	97.9	73.9	24	47.1	16.6	13.1	1.3
2	96.8	92.5	42.2	36.7	19.5	15.3	1.3
3	98.8	81.7	13.3	58.7	28.6	23.7	1.2
4	98.5	96.7	27.4	56.9	25.3	22.9	1.1
5	98.3	89.7	15.8	53.1	36.2	22.8	1.6
6	98,0	90.1	39.6	46.8	13.7	18.1	0.8
7	94.3	80.7	23	36.3	16.1	16.6	1.0
8	96.4	94.8	29.5	54.9	28	8.4	3.3
9	92.4	94.7	42.3	33.9	15	6.6	2.3
10	95.5	91.1	22.1	59.5	34.9	13.9	2.5
11	92.3	71.6	38	49.9	12	14.2	0.8
12	97.9	79.2	22.1	42.4	47.6	26.7	1.8
13	98.6	84.2	33.2	48.9	34	14.3	2.4
14	96.2	93.5	29.6	44.6	26.6	20.5	1.3
15	93.9	78.2	30.9	40.6	20.6	11.3	1.8
**Average**	**96.4**	**86.2**	**28.9**	**47.4**	**25.0**	**16.6**	**1.6**
**SD**	**2.2**	**8.2**	**9.0**	**8.3**	**10.1**	**5.8**	**0.7**
**Median**	**96.8**	**89.7**	**29.5**	**47.1**	**25.3**	**15.3**	**1.3**

The percentages of each subpopulation are shown per individual, along with the group median values. Markers: CD18 (pan-leukocyte), CD21 (B lymphocytes), CD5 (T lymphocytes), CD4 (helper T lymphocytes), CD8 (cytotoxic T lymphocytes), PI (propidium iodide, used to assess cell viability). The CD4/CD8 ratio is also provided. Data are presented as the mean ± standard deviation (SD) and median for each variable.

### Expression pattern of the CD18 marker on feline lymphocytes

**Figure 1:**
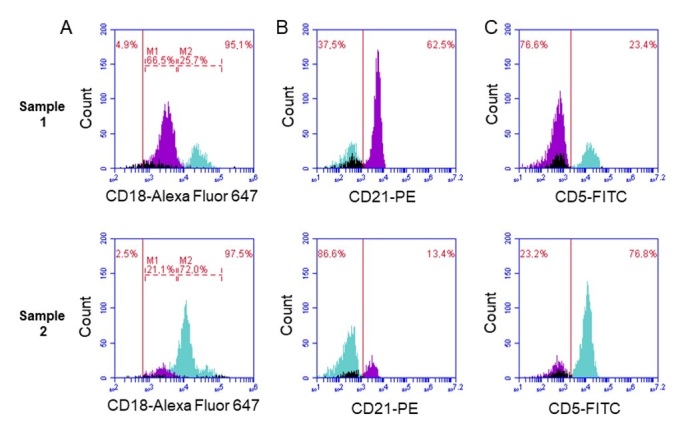
Graphic representation of flow cytometry of two feline peripheral blood samples. (A) Histograms of the labeling with the anti-CD18 antibody conjugated with Alexa 647, where two peaks (sample 1, top) and three peaks (sample 2, bottom) of different fluorescence intensities are observed in the positive population; (B) Histograms of the labeling with the anti-CD21 antibody conjugated to phycoerythrin (PE) corresponding to samples 1 and 2; (C) Histograms of the labeling with the anti-CD5 antibody conjugated to fluorescein isothiocyanate (FITC) corresponding to samples 1 and 2. In all cases, the lymphocyte population is specifically selected, and the B lymphocytes marked in purple (marker M1) and the T lymphocytes marked in green (marker M2) are observed.

When CD18 expression in feline lymphocytes was studied, differential fluorescence intensity was observed among the different cell subtypes, with histograms displaying multiple fluorescence peaks (Figure 1A). To identify the subpopulations corresponding to each peak, Panel 3 (CD18-Alexa Fluor 647, CD21-PE, and CD5-FITC) was used, and the CD21 antibody was replaced with another fluorochrome to avoid fluorescence overlap. This analysis revealed that the peak with the lowest intensity corresponds to B lymphocytes, whereas the peak with the highest intensity corresponds to T lymphocytes (Figure 1).

Interestingly, in some samples, it was even possible to observe three peaks of fluorescence intensity for CD18, where again, the lowest intensity peak corresponded to B lymphocytes and the second and third peaks were associated with T lymphocytes (Figure 1, sample 2). To explore whether these high-intensity peaks reflected differences between CD4+ and CD8+ T-cell subsets within the CD5+ T-cell population, further analysis was conducted. However, no consistent or exclusive correlation was found between specific fluorescence peaks and either T-cell subset.

## Discussion

Two complementary panels (Panel 1: CD18, CD4, and CD8; Panel 2: CD21 and CD5) were employed to perform specific labeling of surface markers, enabling the quantification of lymphocytes and their respective subclasses in 15 cats. Descriptive data were summarized using the median and the minimum–maximum range given the limited sample size. When the total number of CD5+ (T lymphocytes) cells added to the total number of CD21+ (B lymphocytes) cells was analyzed, the values obtained were close to the CD18 values found for all cases. Both are markers of the mature lymphocyte subtype, so minimal differences in the totality of CD18 can be explained by circulating immature cells. However, the addition of CD4+, CD8+ and CD21+ cells is significantly lower, in accordance with what has been reported for healthy cats, where a median of 22.6% of lymphocytes do not express CD4 or CD8 (CD4-/CD8-) (Rout et al., 2020). This may be because these cells are γδT cells, which in cows, sheep, and pigs may constitute the majority of peripheral blood mononuclear cells in young animals. However, the presence of this cell type has not yet been described in cats (Rout et al., 2020), and its characterization could be addressed in future studies.

To our knowledge, there are no published reports on CD18 expression levels in the peripheral blood of healthy cats. However, Rütgen et al. (2022), who analyzed lymph node samples from normal cats (n=11), reported values similar to those reported in our work in peripheral blood (86.93% ± 11.01% *vs.* 86.2% ± 8.2%, respectively). With respect to CD21, the means reported by Vapniarsky et al. (2020) in the peripheral blood of healthy cats (n=8) using the same clone were slightly greater (38.7%) than those of our work. However, Knotek et al. (2000), using other clones for this same antibody in healthy cats (n=36), reported a percentage similar to ours (19.4% ± 9.9%). Furthermore, these authors reported a CD5 value of 65.5% ± 14.4%, which is also consistent with the data obtained in our work. Finally, using the same clones, Campbell et al. (2004) reported an average CD4 concentration of 29.94% and an average CD8 concentration of 19% (n=50), which are in agreement with our observations. In these healthy cats, the CD4/CD8 ratio was similar to that reported internationally (Bęczkowski et al., 2015; Campbell et al., 2004). The limitation of this study is the relatively small sample size. Therefore, additional samples are needed to establish these data as reference values for lymphocytic populations in the peripheral blood of felines. Nevertheless, obtaining baseline information is essential for identifying deviations from normal immunophenotypic patterns, which is crucial for the diagnosis and monitoring of hematopoietic diseases. In this sense, alterations in lymphocyte subpopulations have been reported in feline hematopoietic malignancies and immune-mediated diseases, where immunophenotyping by flow cytometry serves as an essential diagnostic and prognostic tool (Araghi et al., 2014; Guzera et al., 2016). Moreover, deviations in the CD4/CD8 ratio are associated with viral infections such as FIV/FeLV and other immunological disorders, reflecting immune status and disease progression (Bęczkowski et al., 2015; Jaroensong et al., 2022; Litster et al., 2014). Establishing baseline values in healthy cats, as presented here, is fundamental for interpreting immunophenotypic alterations in clinical pathology and advancing feline hematopathology research.

In this study, lymphocyte marker expression was analyzed in fifteen clinically healthy cats of undefined breeds, including both sexes, with a median age of 4 years. Analysis of lymphocyte populations revealed that, in general, the relative percentages of CD5+ T cells, CD8+ T cells, and CD21+ B cells were maintained across the age range, whereas the percentage of CD4+ helper T cells tended to decrease in older cats, resulting in a lower CD4/CD8 ratio (Campbell et al., 2004). Although our study was not designed to specifically evaluate age as a variable, our dataset includes animals of different ages, raising the possibility that part of the biological variability observed could be related to this factor. Future studies specifically addressing age-related changes in feline lymphocyte subsets would be valuable for refining reference intervals and improving interpretation in both clinical and research settings.

When CD18 expression in feline lymphocytes was studied, differences in fluorescence intensity were observed among the different cell subtypes. The identification of the subpopulations revealed that the lowest fluorescence intensity corresponded to B lymphocytes, whereas the highest intensity corresponded to T lymphocytes. This difference in expression, which could be verified for the first time in felines, was previously suggested by Martini et al. (2020), although their findings were inconclusive because of the conflicting combinations of antibodies and fluorochromes used in their work. These findings suggest that CD5+ T lymphocytes in felines exhibit varying levels of CD18 expression. Comparable results have been reported in dogs, where distinct fluorescence intensities were also observed between B and T lymphocyte subpopulations (Comazzi et al., 2006), supporting the findings of the present study. The biphasic pattern of CD18 expression identified in this study may enhance the discrimination between neoplastic and reactive lymphocyte populations, potentially improving diagnostic accuracy in feline lymphoma and leukemia cases where clonal populations are expected. In neoplastic populations, which represent a clonal expansion of lymphocytes, all cells share the same phenotype and therefore display a single CD18 peak. In contrast, reactive hyperplasias involve the simultaneous expansion of multiple lymphocyte subsets with variable levels of CD18 expression, allowing their differentiation from neoplastic populations.

## Conclusions

An immunophenotypic analysis of peripheral blood from healthy cats revealed that viable leukocytes (CD18+) accounted for a median of 89,7% of cells, with CD5+ T lymphocytes being the predominant subset, followed by CD21+ B lymphocytes. Among T cells, CD4+ helper cells were more abundant than CD8+ cytotoxic cells were, with variable CD4/CD8 ratios. Furthermore, the biphasic expression pattern of CD18, with distinct fluorescence intensities for B and T lymphocytes, highlights the complexity of feline immune cell populations. These baseline data are essential for improving diagnostic and monitoring approaches for feline hematopoietic diseases.
